# Predicting Academic Performance: Analysis of Students’ Mental Health Condition from Social Media Interactions

**DOI:** 10.3390/bs12040087

**Published:** 2022-03-23

**Authors:** Md. Saddam Hossain Mukta, Salekul Islam, Swakkhar Shatabda, Mohammed Eunus Ali, Akib Zaman

**Affiliations:** 1 Department of Computer Science and Engineering, United International University, Dhaka 1212, Bangladesh; saddam@cse.uiu.ac.bd (M.S.H.M.); swakkhar@cse.uiu.ac.bd (S.S.); akib@cse.uiu.ac.bd (A.Z.); 2 Department of Computer Science and Engineering, Bangladesh University of Engineering & Technology, Dhaka 1000, Bangladesh; eunus@cse.buet.ac.bd

**Keywords:** psychological attributes and mental health, Facebook, word embedding, MPNet, BiLSTM, regression, classification, ensemble

## Abstract

Social media have become an indispensable part of peoples’ daily lives. Research suggests that interactions on social media partly exhibit individuals’ personality, sentiment, and behavior. In this study, we examine the association between students’ mental health and psychological attributes derived from social media interactions and academic performance. We build a classification model where students’ psychological attributes and mental health issues will be predicted from their social media interactions. Then, students’ academic performance will be identified from their predicted psychological attributes and mental health issues in the previous level. Firstly, we select samples by using judgmental sampling technique and collect the textual content from students’ Facebook news feeds. Then, we derive feature vectors using MPNet (Masked and Permuted Pre-training for Language Understanding), which is one of the latest pre-trained sentence transformer models. Secondly, we find two different levels of correlations: (i) users’ social media usage and their psychological attributes and mental health status and (ii) users’ psychological attributes and mental health status and their academic performance. Thirdly, we build a two-level hybrid model to predict academic performance (i.e., Grade Point Average (GPA)) from students’ Facebook posts: (1) from Facebook posts to mental health and psychological attributes using a regression model (***SM-MP*** model) and (2) from psychological and mental attributes to the academic performance using a classifier model (***MP-AP*** model). Later, we conduct an evaluation study by using real-life samples to validate the performance of the model and compare the performance with Baseline Models (i.e., Linguistic Inquiry and Word Count (LIWC) and Empath). Our model shows a strong performance with a microaverage f-score of 0.94 and an AUC-ROC score of 0.95. Finally, we build an ensemble model by combining both the psychological attributes and the mental health models and find that our combined model outperforms the independent models.

## 1. Introduction

People’s psychological attributes and mental health issues can greatly influence their working performance in real life [1,2,3]. Psychological attributes [4] are essential properties of an individual which play vital role during her interactions in a society. Personality traits [5] are one of the most important psychological attributes which reflect one’s patterns of thoughts, behavior, and feelings. People may differ from each other in terms of personality traits because these traits might vary over time and across situations [6,7]. On the other hand, mental health is an integral part of overall health, which includes our emotional, psychological, and social well-being [8]. Mental health issues impact one in four people worldwide, yet access to care is challenging for those who suffer from them [9]. These illnesses are complex and can take many forms. There is a direct correlation between mental and physical health [10]. Several studies [11,12,13,14,15] have also described that psychological attributes and mental health significantly influence one’s performance in different real-life activities. A few studies [16,17] show strong association with students’ drop out rate and their mental health problems.

Similarly, Zhao et al. [11] show that personality traits and work performance have a strong relationship. Personality influences our eating habits and food choices [12], artistic and scientific creativity [13], and career selection [18]. Education psychology studies [1,19] also show that personality and self-efficacy have effects on students’ academic achievements. A more recent study [3] shows that depression and stress greatly affect students’ academic performance. In this paper, we predict students’ academic performance from their psychological attributes and mental health issues derived from Facebook interactions.

Pennebaker et al. [20] find that what people say and write tend to give information about their actual behavior. This finding indicates that what people write on social media may reveal their actual behavior. In fact, Back et al. [21] empirically showed that people tend to reflect their actual personality and behavior during Facebook interactions. Prior studies [22,23,24] also explored the association between mental health and social media interactions. For example, Choudhury et al. [22] examined the relationship between users’ depression and their Twitter social media platform interactions. In another study, Peng et al. [23] investigated the user’s mental health from profile details, behavioral attributes, and situation described in social media. However, we have found no prior study that made a bridge between students’ psychological and personality traits from the actual social media posts and academic performance.

**Figure 1:**
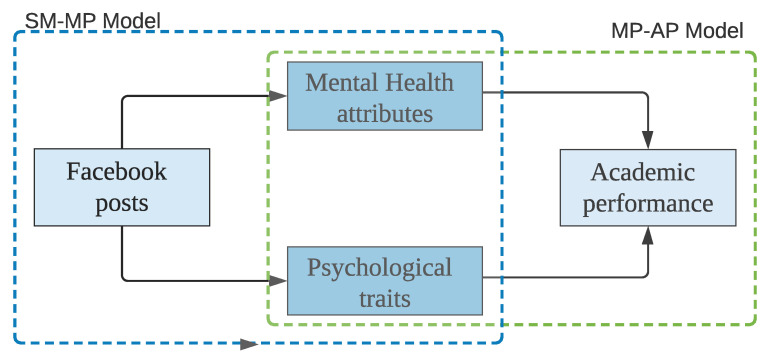
High-level architecture of the academic performance prediction model.

In our study, we collect data of a total of 302 Facebook users. Then, we build a two-level hybrid prediction model to predict the level of academic performances (i.e., high, medium, or low) of students from their Facebook posts. In the first level, we extract 27,889 student posts to analyze their psychological attributes and mental health statuses. We also conduct a survey to collect the ground-truth data of Personality, Self-Efficacy, Depression, and Stress. Next, we build a non-linear regression model by using Bidirectional Long Short-Term memory (Bi-LSTM). In the second level, we evaluate four classification models comprised of one of the conventional multi-class models (K-Nearest Neighbors), one of the tree-based ensemble models (Random Forest), and two of the boosting-based ensemble models (i.e., adaptive boosting (AdaBoost) and light gradient boosting (LgBoost)) using the performance parameters (microaverage of recall, precision, and f-score). Figure 1 shows a high-level architecture of our prediction model. Here, the ***SM-MP*** (social media to mental/psychological) model represents the first level of prediction, and the ***MP-AP*** (mental/psychological to academic performance) model shows the second level of prediction. Finally, we develop a hybrid ensemble model through the stacking of K-Nearest Neighbors, Random Forest, and LgBoost models using a balanced weight distribution. Furthermore, we evaluate the developed models with 70 test participants to validate the accuracy of prediction.

In summary, we provide the following contributions:

- We conduct multi-disciplinary research that bridges between computer science and cognitive science.
- We build a new dataset that comprises students’ psychological attributes and mental health status (i.e., personality, stress, self-efficacy, and depression) and their academic performances, i.e., GPA.
- We build a two-level hybrid prediction model with a fusion of regression (level-1) and classification (level-2) that predicts students’ academic performances from their Facebook posts.

## 2. Background

Mental characteristics and personality traits may play an important role in how people make their decisions and perform an action in their real life. Personality traits are innate characteristics that guide people to behave in a certain way [25]. Furthermore, mental health disorders such as depression, anxiety, and stress may influence their performance. Therefore, we describe these concepts and related works on how social media can help detect these traits.

### 2.1. Depression

Depression is a common psychological disorder among undergraduate students and adults, characterized mainly by the persistence of sadness, feeling blue, and a loss of interest in daily activities [26]. According to Bondolfi et al. [27], depression makes people vulnerable to mental and physical health problems. Untreated depression is a common cause of suicide. According to the Substance Abuse and Mental Health Services Association (SAMHSA) (https://www.samhsa.gov/, accessed on 11 January 2022), 90% of the suicide cases are because of severe depression. People may encounter depression for several reasons. A person may feel depressed due to the death of an intimate person or loosing his job. An individual may feel depressed, despite that she may pass smooth life. Genetic behavior and environmental conditions may also trigger depression. The *Major Depression Inventory* (MDI) [28] is an approved scale to measure the level of depression of an individual. This inventory contains a total of 10 questions. The answer options for these questions range from 1–6 (Likert type scale). These questions ask users how they have felt in the last two weeks. For example, *Have you ever felt low spirit or sad; Have you felt very restless; and Have you had trouble sleeping at night*, are asked to the users.

### 2.2. Stress

Stress is the general reaction when any type of change occurs, which requires an adaption or response. McEwen et al. [29] find that the brain is the central organ of stress because brain points the threatening situations and stores them, which influences the psychological behavior of a person. People react to the changes with emotional, mental, and even physical responses. Even positive changes in our life can lead to stress. For example, a promotion at a job or giving birth to a child can significantly produce stress. If stress continues without any relief in the life of an individual, that may lead to distress. Moreover, distress may lead to headache, improper sleeping, and sexual dysfunction. Therefore, a person can commit self-injuries, suicide, or experience other harmful events. Toussain et al. [30] examine 148 young adults to find out the long-term effects of stress. They reveal that long-term stress may lead to worse psychological and physical health conditions. Cohen [31] designs a 10-item *Perceived Stress Scale (PSS)* scale. Respondents answer in a *Likert* scale of 1–5 for the PSS. These questions ask users about their feelings and thoughts during the last month. For example, the users are asked: *How often have you felt nervous and stressed? how often have you felt that things were not going your way? How often have you been able to control irritations in your life*?

### 2.3. Personality

Personality is a set of inherent characteristics that uniquely distinguish an individual from others. Azucar et al. [32] show that the Big Five model represents the overall personality traits of an individual. Big Five traits are stable for a person, or they may change slightly over the course of time. However, we may notice a change in personality traits due to a traumatic or life-changing experience. According to the Big Five model [33], there are five different traits: *openness, extroversion, conscientiousness, neuroticism, and agreeableness*. We describe briefly the Big Five traits as follows:

- *Openness to experience*: People who usually have strong imagination and who are eager to learn new knowledge generally possess a high score in openness. A person with a high openness score is usually insightful and imaginative.
- *Conscientiousness*: People who have a high conscientiousness score are generally organized and self-disciplined.
- *Extroversion*: The extroversion trait indicates being energetic, outgoing, and social. Extroverted people generally prone to sharing their experiences with others.
- *Agreeableness*: Agreeable people are likely to be friendly, compassionate, kind, and sympathetic with others. They also show kindness, affection, and warm behavior to others.
- *Neuroticism*: People with a high neuroticism score are generally emotionally unstable, nervous, sensitive, and moody.

The *10-Item Personality Inventory (TIPI)* (http://gosling.psy.utexas.edu/scales-weve-developed/ten-item-personality-measure-tipi/ten-item-personality-inventory-tipi/, accessed on 11 January 2022) has been widely used to measure personality. This inventory has 44 statements, and the answer options are in a *Likert* scale of 1 to 5. Some example statements are: *I see myself as Dependable, self-disciplined; I see myself as sympathetic, warm; I see myself as disorganized, careless.*

### 2.4. Self-Efficacy

Self-efficacy [34] refers to an individual’s belief in his or her capacity to execute the behaviors necessary to produce specific performance attainments. A person with high score in *self-efficacy* has a strong judgmental process to execute a course of action to accomplish tasks. Ebstrup et al. [35] outline the 10-item psychometric scale, namely, the *General Self Efficacy (GSE)* scale designed to evaluate the optimistic self-confidence. The items explicitly refer to personal agency, *i.e., the belief that one’s actions are responsible for successful outcomes*, and has a *Likert* scale of 1–4 to answer. Some example statements are: *I can always manage to solve difficult problems if I try hard enough; It is easy for me to stick to my aims and accomplish my goals; I can solve most problems if I invest the necessary effort.*

### 2.5. Context-Based Word Embedding

Context-based Word embedding is the vector representation of a particular word in a document. The technique is capable of capturing the context of a word in a document in terms of semantic and syntactic similarity and its relation with other words. MPNet [36] is a pre-training model that combines masked language modeling (MLM) used in BERT [37] and permuted language modeling (PLM) used in XLNet [38] to convert sentences and paragraphs into a dense 768-dimensional vector space while capturing semantic information. Though XLNet addresses the limitation of neglecting dependency among the predicted tokens in BERT, it cannot leverage the full position information of a sentence, which results in the position discrepancy between pre-training and full-tuning. MPNet achieves the dependency among predicted tokens through permuted language modeling (vs. MLM in BERT) and takes auxiliary position information as input to make the model see a full sentence, thus reducing the position discrepancy (vs. PLM in XLNet) [36].

## 3. Related Work

We observe several studies [22,39,40] that describe the prediction of people’s mental health status from their social media posts. For example, Choudhury et al. [22] predict depression based on the data extracted from social media platform, Twitter. The authors analyze linguistic cues and signals from users’ text by using SVM classifier. In another study, Choudhury et al. [40] predict different types of emotions by analyzing text from Twitter posts. They collect the ground-truth data of Twitter users on depression by crowd-sourcing. They build an SVM classifier to predict depression with a high level of accuracy (73%). Park et al. [39] find that normal individuals exploit Twitter for gaining information, while depressed individuals use it as an instrument for social consciousness and emotional interchange. Tan et al. [41] find a relationship between users’ value and behavior to visit national park services among a total of 413 tourists.

Peng et al. [23] conduct research where they investigate users’ mental health conditions by using their micro-blog texts, profile details, and behavioral attributes extracted from their social media. They build multi-kernel SVM models to choose the optimal kernel for individual attributes in order to determine the depressed user. They compare their model with single-kernel SVM [42], KNN [43], Decision Trees [44], Naive Bayes [45], and a multi-kernel SVM model [46] for classifying depressed people with a reduced error rate to 16.54%. Dinakar et al. [47] study teenagers suffering from depression who seek help on the Internet. They build a machine learning model for investigating the main factors for stress of teenagers. They use supervised and unsupervised models for predicting depression. Choudhury et al. [48] predict human effective states from their social media data.

Pak et al. [49] collect a total of 300,000 tweets that are divided into 3 sets of documents: *negative, positive*, and *factual*. TreeTagger classifier is then used based on the multinomial Naive Bayes classifier, which uses *N-gram* and *POS* tag features. Gao et al. [50] show a computational model to predict a user’s attitude in terms of sensation, opinion, and probability of action on contentious social media subjects. We also observe a few studies [22,51] that focus on defining the mental and behavioral changes of new mothers in prenatal postpartum phases using their tweets. The researchers predict activity, emotion, and linguistic style measurements by three different classifiers. In another study on stress [52], the authors investigate post-traumatic stress disorder, a severe disease influencing millions worldwide, especially among military professionals.

In the light of the above discussion, we find that none of the previous studies have been conducted to predict users’ academic performance from their psychological attributes and mental health status derived from social media. We build a two-level hybrid classification model with the fusion of classification and regression models. The regression model generates the mental health and psychological attributes at the first level using the Facebook posts of an individual student. Then, a hybrid ensemble classification model predicts the academic performance of that student. To the best of our knowledge, our work is the first study that exploits semantic relationships (i.e., MPNet) from Facebook statuses to predict users’ academic performance.

## 4. Methodology

In this section, we describe the different steps of our proposed technique. First, we conduct a survey to assess psychological and mental health conditions of users. After that, we collect the Facebook interactions (i.e., posts) of these users. Finally, we build the prediction model for Facebook interactions and psychological attributes and mental health status. Towards this direction, we perform the following steps:

- *Collecting and pre-processing dataset.* We collect the users’ textual content using the downloaded dataset provided by each individual user. Users were contacted virtually, and they provided their dataset themselves.
- *Feature selection.* We find two different levels of correlations: (1) users’ social media usage and their psychological attributes and mental health status and (2) users’ psychological attributes and mental health status and their academic performance i.e., GPA. We use *Pearson’s correlation coefficient* [53] to select the significant features for psychological attributes and mental health status and GPA.
- *Building model.* We build a two-level hybrid model to predict the GPA from a student’s Facebook posts: (1) from Facebook posts to mental health and psychological attributes (***SM-MP*** model) and (2) from psychological and mental attributes to academic performance (***MP-AP*** model). For the sake of brevity, throughout this paper, we mention the models of *Social Media to Mental and Psychological attributes* and *Mental and Psychological attributes to Academic Performance* as the ***SM-MP*** and ***MP-AP*** models, respectively..
- *Model validation.* We investigate our ***SM-MP*** model, and find the *R^2^* as 35.21%. In the ***MP-AP*** model, we achieve an AUC-ROC score of 0.95. We also test our method using a real-life test case, which shows the effectiveness of our proposed approach.

### 4.1. Collecting and Pre-Processing Dataset

We invited a total of 357 students from the Faculty of Science, Engineering, and Economics of United International University (https://www.uiu.ac.bd/, accessed on 11 January 2022) to participate in our research. Among them, 25 students did not show interest to attend the survey and 30 students did not agree to share their Facebook data. Thus, with the remaining 302 students (male = 178, female = 124), we conduct the survey in person or by using a *Google Form*. These students also provide us their extracted Facebook posts. Table 1 presents the statistics of students’ Facebook posts.

We recruited students by using the *judgmental sampling* [54] technique. Since participants needed to attend our study carefully in several surveys (i.e., MDI, PSS, IPIP, and GSE) and share their Facebook statuses, we conveyed that this study is going to investigate the relationship between students’ academic performance and their psychological attributes and mental health. They understood the necessity of this study and attended this study with interest and sincerity. Thus, we consider that a non-probability sampling such as judgmental sampling is the reasonable technique in our context [55]. We obtained ethical approval from the Institute of Advanced Research (IAR) (https://iar.uiu.ac.bd/, accessed on 11 January 2022) of the university. We explained the purpose of the study to the participants and also mentioned that their data will be securely stored and will not be distributed to others. We also assured that the data will be anonymous before publishing the results of the survey.

We collected users’ data in two steps. In the first step, we conducted a survey to measure users’ psychological attributes and mental health status. We performed surveys in either the on-site or online modes to collect the ground truth data. In our questionnaire, we had a total of 74 items: for stress (10 items), depression (10 items), self-efficacy (10 items), and *Big Five* personality (44 items). In the second step, we requested the users to provide us with their Facebook posts by downloading them as a compressed folder from the settings option of their Facebook profiles. The Facebook data were collected on April 2021, and due to the pandemic situation, the users were contacted through virtual meetings or phone calls. Additionally, with due permission from the students, we collected their academic results (i.e., GPA of Spring 2021). We computed the *Cronbach’s alpha* test of users’ answers for the psychological and mental health questionnaire. Cronbach’s alpha [56,57] estimates the internal consistency of reliability of test scores. We found the score of the tests to be in the range of 0.50–0.65; the study of Chen et al. [56] shows that these scores are acceptable.

We have utilized the Facebook dataset from those users who attended the survey and provided us with their Facebook posts. We pre-processed these posts using regular expressions to remove all the *URLs*, default “*message*“ tags, punctuation marks, unnecessary white spaces, and empty strings. Our data contained a few local languages. We detected non-English languages by using a python library *TextBlob* (https://textblob.readthedocs.io/, accessed on 11 January 2022) and discarded those contents. We also used a custom dictionary to convert the short form of a regular word into a typical word. For example, “n8“, “bro“, “m“, “u“ are converted to “night“, “brother“, “am“ and “you“, respectively. Finally, we used the popular *spaCY* (https://spacy.io/, accessed on 11 January 2022) library “*Lemmatizer*“ to lemmatize the text posts.

### 4.2. Feature Selection

In this section, we describe the techniques of how we selected the relevant features for building our model. Students’ Facebook posts contain textual content. To feed these data, we converted the text into vector representations using sentence transformer (MPNet) [36], a context-based word-embedding technique. We used MPNet because it achieves the dependency among predicted tokens through permuted language modeling (vs. MLM in BERT) and takes auxiliary position information as input to make the model observe a full sentence, which reduces the position discrepancy of the model (vs. PLM in XLNet). As we used a context-based word-embedding technique, additional feature selection methods were not required. In the second level, we identified important features to predict users’ academic performance from their psychological attributes and mental health status. Since both of the dependent (i.e., academic performance) and independent (i.e., Big5, Self-Efficacy, Depression, and Stress) variables were continuous, we used Pearson’s correlation coefficient. We selected relevant attributes that were strongly related to our dependent variable (i.e., GPA). Table 2 presents the correlation between psychological attributes and mental health status and academic results (i.e., GPA).

### 4.3. Building Models

In this section, we describe the process of building our models. In the first step, we built a regression model from the social media dataset to mental health and psychological attributes (***SM-MP*** model). In the next step, we built a classifier model to predict the students’ academic performance levels (High/Medium/Low) from their psychological attributes and mental health status (***MP-AP*** model).

#### 4.3.1. ***SM-MP*** Model

After cleaning the texts, we applied MPNet by using Python’s SentenceTransformers framework (https://www.sbert.net/docs/pretrained_models.html, accessed on 11 January 2022. We imported one of the versions of MPNet named all-mpnet-base-v1 (https://huggingface.co/sentence-transformers/all-mpnet-base-v1, accessed on 11 January 2022) as the pre-trained model base and extracted a dense 768-dimensional vector space while capturing the semantic information for the input text. We saved the vectors as a pickle file in our local Google drive.

**Figure 2:**
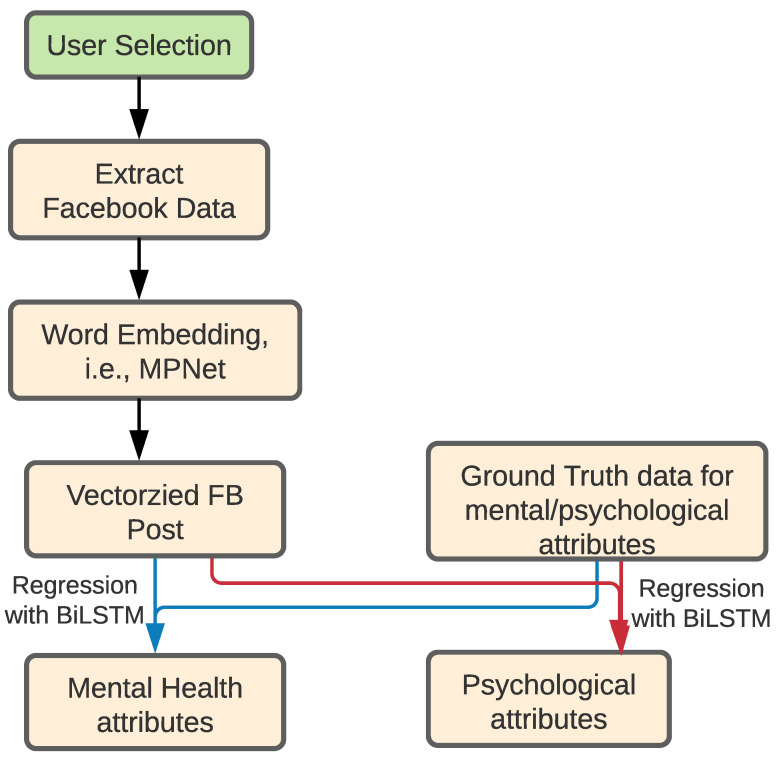
Architecture of predicting mental health and psychological attributes (***SM-MP***) from Facebook posts.

We fed the dataset, which had a dimension of 44,951,024, to the word-embedding layer. In our Facebook dataset, we found that an individual student had a total of 4495 maximum words in his or her statuses. Each word is represented by 768 vectors. Then, the output was fed to a BiLSTM layer having 64 neurons with the return sequence set as True. The BiLSTM gave an output of (None, None, 128) dimension, and it contained 557,568 parameters. We used the Dropout [58] technique for regularization to avoid overfitting during training phase of the neural network. A dropout layer does not change the dimensions of the data, and it has zero parameters. We fed the output to a new BiLSTM layer having 32 neurons. This layer gave an output shape of (None, 64), and the number of the parameters was 41,216. Then, the output was fed to 3 different dense layers with 256, 128, and 1 neurons, respectively. We used mean square error [59] for loss function and Adam with learning rate = 0.0005 as an optimizer. We used *tanh* [60] as an activation function for the hidden layers. For the output layer, we used Linear [60] activation function. Figure 2 demonstrates the architecture of predicting mental health and psychological attributes from Facebook posts.

Table 3 shows that our BiLSTM model demonstrates the best performance among the independent models. We used *mean square error* [61] for loss function and *Adam* with *learning rate = 0.001* as an optimizer. We executed our model with distinct values to find the value that shows the best performance while keeping the training time to a minimum. We executed the models with smaller values of 0.01 and 0.001. We used *tanh* [60] as an activation function. The primary benefit of using *tanh* is that it is zero-centered, which made it easier to model inputs that had negative, neutral, and positive values. We did not use *Softmax* because, typically, it is used for multi-class classification with cross entropy loss function, while we compute non-linear regression with *mean square error* as loss function.

The *Sigmoid* was not used because it is not zero-centered, and it gives output values between 0 and 1. We did not use *ReLU* because of the *Dying ReLU* issue, where some *ReLU* neurons fundamentally die for all inputs and stay inactive regardless of the input value. We found from different models (see Table 3) that self-efficacy shows the strongest potential (R^2^ 0.35), while neuroticism shows the weakest potential (R^2^ 0.19).

#### 4.3.2. ***MP-AP*** Model

After selecting the relevant features, we built a multi-class classifier model (***MP-AP*** model) from users’ psychological attributes and mental health status to their academic performance. The outputs of the psychological attributes and mental health status from the ***SM-MP*** model were used as the independent features, whereas the level of academic performance was used as the dependent feature in terms of three different labels: low (less than 2.5), medium (between 2.5 to 3.5), and high (greater than 3.5).

We compared four classification models to find out the best-performing model for predicting the academic performance. One conventional multi-class model (K Nearest Neighbors), one tree-based ensemble model (Random Forest), and two boosting-based ensemble models (AdaBoost and LgBoost) were evaluated to find out the best-performing model. We evaluated the models using an 80/20 train–test split by using the extracted output of the mental health and psychological features. Being a multi-classification problem, the performance of the models were compared using the microaverage of the f-score and area under the receiver operating curve (AUC-ROC).

**Figure 3:**
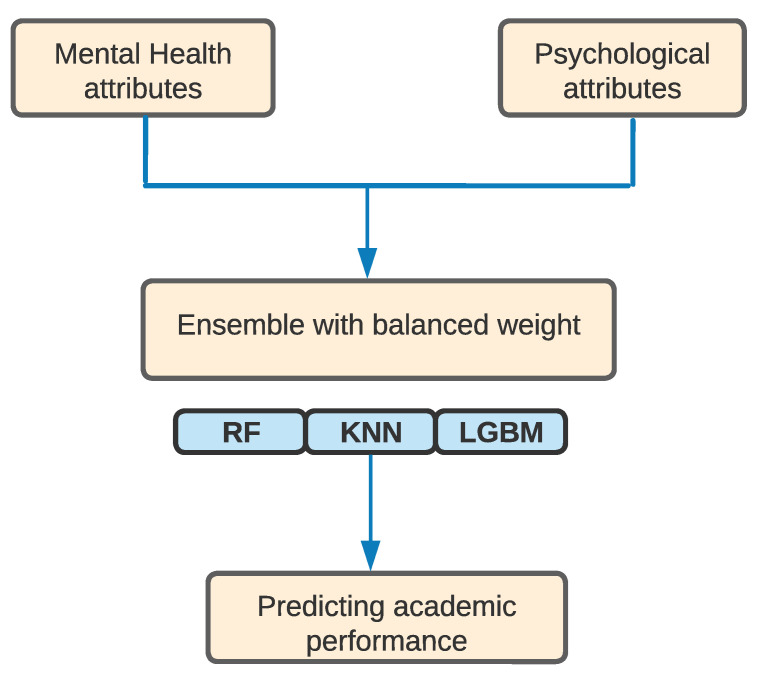
Architecture of predicting users’ academic performance (i.e., high, medium, and low) from mental health and psychological attributes (***MP-AP***).

**Figure 4:**
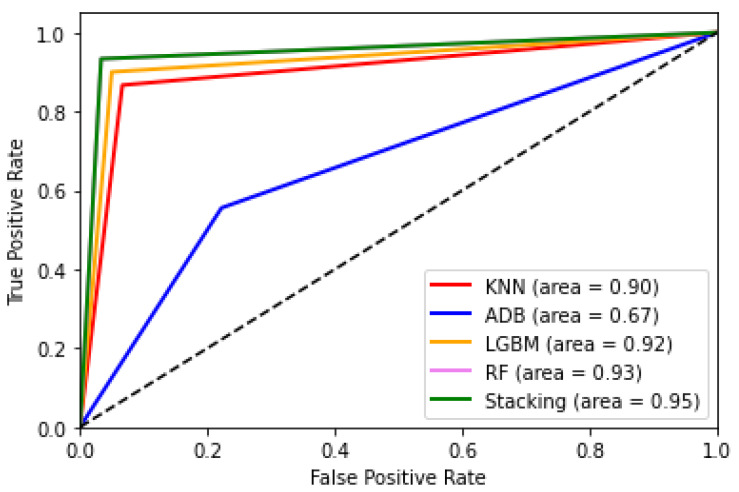
Comparison of different models using AUC-ROC.

Among the models, AdaBoost performed the poorest, with a score of 0.69. In contrast, KNN showed moderate performance with a score of 0.87, whereas Random Forest and LgBoost outperformed those models with scores of 0.91 and 0.89, respectively. Furthermore, we developed a hybrid ensemble model through the stacking of KNN, Random forest, and LgBoost using balanced weights, as shown in Figure 3. Table 4 shows the performance of the models measured using the microaverage of f-score of the test samples. The developed multi-variant ensemble model outperformed the previously discussed models with an f-score of 0.94, which signifies the superiority of the model with an increase of 0.03 in terms of f-score from the second best-performing model (RF). Additionally, the model demonstrated an AUC-ROC score of 0.95 (see Figure 4). With a score of 0.93, Random Forest performed closest to the developed ensemble model, and AdaBoost performed the poorest with a score of 0.67.

### 4.4. Evaluation

In this section, we validate the developed models by collecting the actual psychological attributes and mental health status and level of academic performances of the students who were not included in the data collection procedure and comparing these data with the prediction of the developed models. For the validity of our hypothesis test, we used *Chi-square* and *Paired t* tests. The tests actually compared the difference between our predicted and actual psychological attributes, mental health, and academic performance results. We conducted the validity tests of the above hypotheses [57,62]. We investigated the performance of the ***SM-MP*** model using *Paired t* test in case of predicting the psychological attributes and mental health status of a student from his Facebook post. On the other hand, we applied *Chi-square* tests to understand the validity of the ***MP-AP*** model by using the using the explored psychological attributes and mental health status in the previous step. Additionally, we compared the performance of the developed model with two of the baseline models using the Average R^2^ (in case of ***SM-MP***) and AUC-ROC (in case of ***MP-AP***) analysis of the test data.

#### 4.4.1. Validation through Comparing

**Figure 5:**
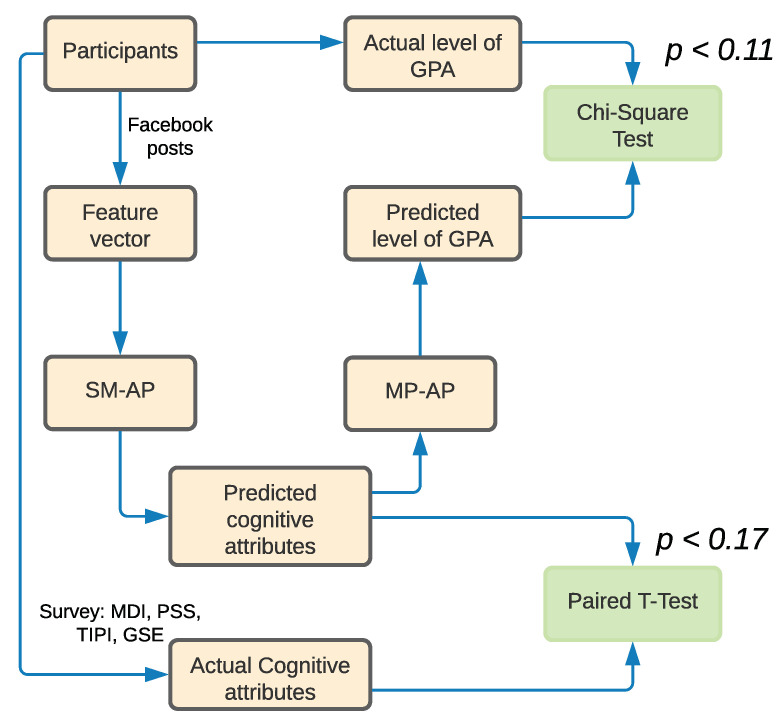
Design of the evaluation study to validate the model using real-life samples.

Our step-by-step validation process has been described in Figure 5. We first obtained a total of 70 university students’ GPA to validate our prediction method and labeled them as high (greater than 3.5), medium (between 2.5 to 3.5), and low (less than 2.5). We present the statistics of the collected data from users in Table 5. We collected Facebook posts of these 70 students by using the Graph API explorer. Additionally, we collected the actual scores of these psychological attributes and mental health status by using various surveys (MDI, PSS, TIPI, and GSE). Then, we applied Sentence Transformer (MPNet) model over their Facebook posts. Later, we fed the feature vectors into the SM-AP model (Level 1) and predicted the scores of psychological attributes and mental health scores. We compared the actual values of the psychological attributes and mental health status and predicted the scores of the psychological attributes and mental health status. Then, we conducted *Paired t* tests [63] to check if the means of these sets of psychological attributes and mental health statuses are significantly different by using IBM SPSS. The results showed that the *p*-value was <0.17 which is not statistically significant. The result of the *Paired t* test indicated that our hypothesis accepts the null hypothesis (i.e., rejects the alternate hypothesis), which claims that there is no difference between the actual and predicted level of students’ results. Then, we fed the relevant features, i.e., Depression, Self-efficacy, Conscientiousness, and Agreeableness (see Table 2), to the ***MP-AP*** (Level 2) model to predict the level of academic performance of the participants. We compared the actual level and the predicted level of academic results to validate the performance of the model by using *Chi-Square test* [64], since both of the levels are categorical in nature. Then, we obtained that the *p*-value was <0.11, which also established that our hypothesis accepts the null hypothesis (i.e., rejects the alternate hypothesis). In summary, we can claim that our predicted and actual level of academic performances have no statistical difference.

#### 4.4.2. Comparison with the Baselines

For the first baseline, we assumed that the academic performance can be predictable with the Linguistic Inquiry and Word Count (LIWC)-based technique. We selected LIWC as a baseline model because the technique has been proven successful in analyzing and predicting users’ psychological states from their word usage pattern [65,66]. We also analyzed our text by using *Empath* [67], for converting users’ posts into lexicon-frequency-based approach. *Empath* generates new lexical categories on demand.

**LIWC-based performance prediction (Baseline 1)**: LIWC is a psycho-linguistic tool for text analysis. LIWC 2007 identifies 74 different features from the text data into different categories, where each category contains hundreds of words [68]. These categories include standard counts, psychological processes, relativity, personal concerns, and other dimensions. We first analyzed the Facebook posts of the 70 students by using LIWC. Then, we identified relevant features from 74 categories, where LIWC categories are independent variables, and users’ psychological attributes and mental health statuses are dependent variables. We used R Leaps package (https://cran.r-project.org/web/packages/leaps/leaps.pdf, accessed on 11 January 2022) using an efficient branch-and-bound algorithm to find the best subset of LIWC categories. By using these relevant features, we built our regression model for predicting psychological attributes and mental health status. From the psychological attributes and mental health status that we predict from the LIWC categories, we next predict users’ level of academic performance. Empirical results using LIWC showed their ability to detect context in a wide variety of experimental environments, including concentration, emotionality, social relationships, modes of thought, and individual differences.

**Empath (Baseline 2)**: We also analyzed our evaluation dataset by using *Empath* [67]. Empath is a deep-learning-based psycho-linguistic tool that produces and validates new lexical categories on demand from a small set of seed words (such as “bleed“ and “punch“ to produce aggression in the category). Empath draws connotations between words and phrases by neural embedding across more than 1.8 billion words of modern fiction. It uses its neural embedding to discover new related terms and then validates the category with a crowd-powered filter. It also analyzes text through 200 built-in, pre-validated categories, such as neglect, state, created from common topics in our dataset. We again identified the best subset of features that were correlated with users’ psychological attributes and mental health statuses through R Leaps implementation package. Finally, we predicted students’ class of academic performance from the scores of correlated psychological attributes and mental health statuses. Table 6 shows the performance of different models.

## 5. Discussion

In this paper, we build a two-level hybrid model to predict students’ academic performance, from students’ actual Facebook posts. We utilize social media posts to calculate psychological attributes and mental health status of students. Thereafter, we predict academic performance from these psychological attributes and mental health status. Therefore, our study integrates different psychological attributes and mental health status derived from Facebook interactions to predict students’ academic performance. In the first level, we exploit users’ Facebook posts to investigate their mental and psychological status. We identify the semantic relationships among the words by analyzing words from left–right and right–left according to the context. We perform the context-based word embedding by using the Sentence Transformer (MPNet) word-embedding vector, where we use a deep-learning-based BiLSTM model. In the conventional methods, researchers usually identify important features, but the sentence transformer model (MPNet) usually considers the whole content as an individual vector. Note that MPNet has a vector size of 768. Since the associations between different variables are non-linear in nature, we use non-linear techniques in our research to predict the mental health and psychological attributes. Our results show that the BiLSTM model performs better than the LSTM and GRU models. This is because, both LSTM and GRU use one forward pass and, therefore, cannot capture the relationship among the words in backwards directions. In contrast, BiLSTM can identify relationships among the words in both directions, which produces a more accurate model. Regarding our finding that the BiLSTM model shows better prediction accuracy, we note that prior studies [56,69] also show similar performance results. We observe that our model moderately predicts all the psychological attributes and mental health status. Among these, we observe that the model predicts self-efficacy scores more accurately. In contrast, we also observe that our model predicts neuroticism personality traits poorly. The reason behind this could be that people may not always feel comfortable in writing their negative attitude in social media.

In the second level, we predict students’ academic performance from their psychological and mental health attributes extracted from the previous level.

Gao et al. [70] find that depressive symptoms among Taiwanese adolescents have negative association with their academic performance by conducting a multivariate regression analysis. In the study, authors found that association may be related to the academic stress that is often found in education systems with high-stakes examinations such as Taiwan. In Bangladesh, students in private universities also have three semesters in a year, and in a semester, they have a total of six examinations (four class tests, mid- and final-term exam papers) in a single course, and they take more than three courses on an average, which might arouse depressive symptoms. Students nowadays are also addicted to the Internet and digital devices. Kumar et al. [71] also show that Internet addiction may promote depressive symptoms and impact their academic performance. In our study, we also obtained similar findings, which can be backed by these previous studies [70,71]. In another study [72], we find that students who have a high level of engagement with academic activities have high scores in self efficacy; in our study, we also find that academic performance and self efficacy are positively correlated.

Dogan [73] finds that neuroticism negatively influences academic performance for both short-term examinations (i.e., final exam) and long-term preparation (i.e., final project) among students. For extraversion, they find weak relationships because the graduation project requires a good collaboration with the teacher coordinator and group members. Kim et al. [74] find that conscientiousness is a strong predictor for academic performance. However, authors do not find any connection between openness and academic performance, which is not aligned with our findings. However, we may explain that open people are likely to apply their reasoning and brain stimulus to solve any problems, and they tend to accomplish the task in a crafted and shorter way than that of others. Thus, they might be good performers in academic activities as well.

In contrast, we notice that extroversion trait has negative significant correlation with students’ GPA. Later, we evaluate four classification model comprising one of the conventional multi-class models (K-Nearest Neighbors), one of the tree-based ensemble models (Random Forest), and two of the boosting-based ensemble models (AdaBoost and LgBoost) using the performance parameters (microaverage of recall, precision, and f-score). Finally, we develop a hybrid ensemble model through the stacking of K-Nearest Neighbors, Random Forest, and LgBoost using balanced weight distribution. The developed hybrid model outperform other models with a good AUC-ROC score of 0.95 and an f-score_micro_ of 0.94. According to the study of Zhuang et al. [75], *A/B exam paper* finds the degree of fairness between two sessions (A and B) of exam papers even though the knowledge point is the same. In our study, to maintain the fairness of the survey questionnaire, we provide the same set of questions to all the participants. In addition to the variable selection during our model building, we systematically select statistically significant variables which generate the maximum accuracy. Therefore, we did not use any A/B exam papers in our experiment; rather, we emphasized more on the standardized tests, which were designed by expert researchers.

### Contributions

Our study has two major contributions. First, our work is the first study that integrates different psychological attributes and mental health statuses derived from actual Facebook interactions to predict students’ academic performance. Most prior literature use self-reported survey data to predict students’ academic performance from students’ Facebook activities [76]. In contrast, we build our model using actual Facebook interactions. Furthermore, we also note that much of the prior literature employs self-reported data to predict negative consequences of Facebook use [77,78,79]. Indeed, there are several prior studies [22,39,40] that use actual social media interactions, especially from the Twitter platform. Therefore, our approach of using actual Facebook interactions to predict stress and depression encourages this body of literature to use objective measures more in model building. Second, to the best of our knowledge, our work is the first to build a two-level prediction model with a fusion of regression and classification techniques that predict the level of students’ academic performance from their Facebook posts. In doing so, we propose a novel balanced weighted hybrid ensemble classifier model where we integrate conventional, tree-based, and boosting-based models. Our model shows good prediction capability with an AUC-ROC of 0.95, which signifies a correct prediction in 95% of the test cases. Applications can be built using our approach that may help to detect the students who may dropout in the future. Interventions against dropout can be planned based on such early detection.

## 6. Conclusions

We have built a two-level hybrid prediction model to predict students’ academic performance, i.e., GPA, from their Facebook interactions. In the first level, we have converted students’ Facebook posts by using the MPNet technique. Then, we have built four different types of linear regression models: Big Five Personality, Self-Efficacy, Depression, and Stress from Facebook posts. In the second level, we have built a hybrid ensemble classifier model to predict the level of academic performance of the students from their mental health and psychological attributes. Our models have obtained a strong prediction potential. There are some avenues to extend our research. We plan to augment photo related activities along with the Facebook statuses to understand users’ psychological and mental health attributes better. Several studies [80,81,82] also show that profile photos of Facebook users can also reveal their psychological attributes such as personality traits and interaction style. Study [83] shows that different social media reveal similar types of psychological attributes for an individual user. In this study, we are also interested in predicting users’ academic performance and computing their psychological attributes and mental health from different social media such as Twitter, Reddit, etc. Students’ academic performances may fluctuate [84,85] for different reasons, and we would like to investigate whether users’ psychological and mental health attributes are the vital factors.

## Figures and Tables

**Table 1:** Statistics of Facebook dataset.

Statistics	Size
Number of users/students	302
Total word count	261,969
Average word count	738
Max word of a user	4495
Min word of a user	23
# of posts	27,889
# of average posts	92.35
Max posts of a user	406
Min posts of a user	6

**Table 2:** Pearson’s Correlation coefficients between psychological attributes and mental health status and academic performances. ${}^{*}$ p<0.05, ${}^{**}$p<0.01.

	DI	PSS	GSE	Ex.	Ag.	Con.	Neu.	Op.
**GPA**	−0.197 **	−0.02	0.23 **	−0.09	0.04	0.19 **	−0.14 *	0.28 **

**Table 3:** Strength of the regression models from MPNet to psychological attributes and mental health status.

Model	R^2^ of Cognitive Attributes							
	Mental Attributes		Psychological Attributes					
	Depr.	Stre.	Self-Eff.	Extra.	Agree.	Consc.	Neur.	Open.
**BiLSTM**	0.32	0.29	0.35	0.25	0.20	0.31	0.19	0.28
**LSTM**	0.21	0.35	0.09	0.23	0.09	0.14	0.23	0.08
**GRU**	0.20	0.31	0.15	0.23	0.08	0.05	0.23	0.16

**Table 4:** Comparison of different models using performance parameters.

Models	Precision_micro_	Recall_micro_	F-Score_micro_
KNN	0.87	0.87	0.87
Random Forest	0.91	0.92	0.91
AdaBoost	0.71	0.68	0.69
LgBoost	0.90	0.89	0.89
Hybrid	0.95	0.94	0.94

**Table 5:** Statistics of evaluation dataset.

Statistics	Size
# of users	70
# of users with High Academic performance	23
# of users with Medium Academic performance	22
# of users with Low Academic performance	25
Total word count	55,105
Average word count	787
Max word of a user	3171
Min word of a user	42
# of posts	5250
# of average posts	75
Max posts of a user	354
Min posts of a user	13

**Table 6:** Comparison of our model with the baseline models.

Model	***SM-AP*** (Average R^2^)	***MP-AP*** (AUC-ROC)
LIWC	0.24	0.87
Empath	0.23	0.85
MPNet	0.35	0.95

## Abbreviations

The following abbreviations are used in this manuscript:

***SM-MP***	Social media to mental/psychological attributes
***MP-AP***	Mental/psychological attributes to academic performance
LIWC	Linguistic inquiry and word count
BiLSTM	Bidirectional long short term memory
MDI	Major depression inventory
PSS	Perceived stress scale
TIPI	Ten-item personality inventory
GSE	Generalized self efficacy
GPA	Grading point average
